# Mediation effect of healthy lifestyles on the association of socioeconomic status with mortality among US cancer survivors: a population-based cohort study

**DOI:** 10.1097/JS9.0000000000002904

**Published:** 2025-07-08

**Authors:** Yuhang Shang, Weilun Cheng, Anbang Hu, Zhengbo Fang, Jiangwei Liu, Fei Ma, Baoliang Guo

**Affiliations:** Department of General Surgery, The Second Affiliated Hospital of Harbin Medical University, Harbin, China

**Keywords:** cancer survivors, healthy lifestyles, mediation analysis, NHANES, socioeconomic status

## Abstract

**Background:**

Socioeconomic inequity can lead to health disparities among cancer survivors. Although lifestyle factors are commonly considered as mediators between socioeconomic status (SES) and health, their impacts on SES-related mortality differences among cancer survivors are still unclear. This study aimed to explore the mediation effect of overall lifestyles on the relationship between SES and mortality among cancer survivors.

**Methods:**

This study analyzed data from 2411 cancer survivors in National Health and Nutrition Examination Survey from 2000 to 2013. Deaths were ascertained from linkage to the National Death Index with follow-up until 2019. SES was assessed by latent class analysis using family income, occupation, education and health insurance. A healthy lifestyle score was constructed based on smoking, alcohol consumption, physical activity and diet. Cox regression and mediation analyses were conducted to quantify the proportion of SES-related survival disparities explained by lifestyle factors. Finally, joint associations between SES and lifestyles on mortality risk were evaluated.

**Results:**

During a mean follow-up of 9.0 years, 894 deaths were recorded. Compared with high SES participants, those with low SES had higher risks of all-cause mortality (HR, 2.54; 95% CI, 1.85–3.05), cancer mortality (1.92; 1.13–3.24), and non-cancer mortality (2.92; 1.96–4.33), and the proportions mediated by lifestyles were 13.5% (7.3–23.5%), 28.7% (10.7–57.6%), and 8.7% (3.5–20.3%), respectively. Additionally, joint analysis revealed that individuals with low SES and unhealthy lifestyle behaviors had the highest risk of all-cause and cancer mortality.

**Conclusions:**

Unhealthy lifestyles partially mediated socioeconomic inequity in mortality among US cancer survivors. Additionally, significant joint effects of lifestyles and SES on mortality were found. Therefore, healthy lifestyle promotion is warranted to mitigate SES-related health disparities among cancer survivors.

## Introduction

The global population of cancer survivors has been rising rapidly, with 19.3 million new cases reported in 2020 and an estimated increase to 28.4 million by 2040^[1,2]^. Despite significant advancements in life expectancy among cancer survivors over the past few decades, considerable challenges remain in improving long-term survival^[3]^. Hence, identifying modifiable variables related to survival outcomes in cancer survivors has become a public health priority.

**HIGHLIGHTS**

- Low SES was significantly linked to high mortality risk among cancer survivors.
- Unhealthy lifestyles partially mediated SES-related mortality disparities.
- Mediation proportion of lifestyles for cancer-specific mortality reached nearly 30%.
- SES and lifestyle showed significant joint effects on mortality risk.
- Promoting healthy lifestyles could reduce mortality in cancer survivors.

Socioeconomic status (SES) is a comprehensive indicator representing a person’s social standing based on their education, income, occupation, and living conditions. The related socioeconomic inequality has substantially contributed to disparities in health outcomes among cancer survivors^[4,5]^. Cancer survivors with lower SES face significant disadvantages compared to those with higher SES, including reduced access to advanced therapies and suboptimal cancer care, which contribute to lower survival rates^[6,7]^. A study based on the National Cancer Database found that gastric cancer patients with lower income and education levels were independently associated with higher mortality^[8]^. Additionally, Pageot *et al* reported that lower education level was linked to poor breast cancer-related outcomes and clinical prognosis^[9]^. Disparities in survival outcomes of cancer survivors attributable to socioeconomic inequality are becoming increasingly pronounced, posing a significant burden on public health^[10]^. Therefore, immediate efforts are essential to reduce health disparities arising from socioeconomic inequality among cancer survivors.

Modifiable lifestyle factors including smoking, physical activity, alcohol intake, body weight, and diet quality are often regarded as mediators between SES and health outcomes, with healthy lifestyles alleviating the adverse health effects associated with socioeconomic disparities^[11–13]^. Current evidence suggests that regular exercise, a healthy diet, abstinence from smoking and maintaining a healthy weight significantly reduced mortality risk among cancer survivors^[14–16]^. However, important knowledge gaps remain. First, the mediating role of lifestyle factors in the context of SES-related mortality disparities among cancer survivors has not been comprehensively explored. Second, limited research has explored the joint effects of SES and overall lifestyle patterns with mortality of cancer survivors. Third, previous studies often use single indicators (e.g., income, occupation, education level) or sum of these indicators to represent socioeconomic level, which could not fully reflect overall SES^[8,17]^. A composite SES measurement that reflects multiple socioeconomic factors is needed to characterize.

Therefore, using nationally representative data from the National Health and Nutrition Examination Survey (NHANES), we aimed to evaluate the association between SES, healthy lifestyles, and the mortality risk of cancer survivors and investigate the potential mediating roles of healthy lifestyles. Socioeconomic determinants are challenging to modify in the short term, but lifestyle, as a potential mediator between SES and health outcomes, presents a modifiable target for intervention. Our study provides a new insight into improving survival outcomes for cancer survivors. This cohort study has been reported in line with the STROCSS guidelines^[18]^.

## Methods

### Study population

**Figure 1.**
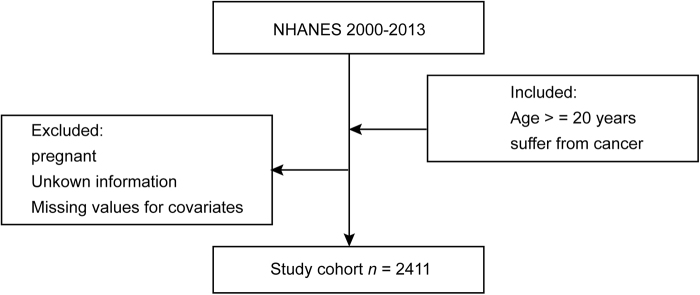
Flowchart of the participants selection from NHANES 2000-2013.

The data of this prospective cohort study were extracted from the 2000–2013 NHANES database (accessed on 15 January 2025). The NHANES database recruited nationally representative health-related data on noninstitutionalized general US civilians, utilizing a complex, multistage probability sampling design, which has been conducted periodically before 1999 and continuously thereafter. All the NHANES protocols were approved by the National Center for Health Statistics Research Ethics Review Board, and written informed consent was obtained from all participants or legally authorized representatives. Data analyzed in this study included demographic data, dietary data, examination data, and questionnaire data. This study included 2411 cancer survivors aged 20 years or older. Participants who were pregnant at baseline, had missing information on cancer investigation, dietary recall, socioeconomic factors, lifestyle factors, other covariates, or incomplete follow-up were excluded from this study. The flowchart of participants’ selection process is summarized in Figure 1. This study was conducted following the guideline of strengthening the reporting of cohort, cross-sectional, and case–control studies in surgery (STROCSS 2025)^[18]^.

### Diagnosis of cancer

Data on the cancer diagnosis were gathered from a self-reported questionnaire “Have you ever been told by a doctor or other health professional that you had cancer or a malignancy of any kind?” Participants were defined as cancer survivors if they responded “yes.”

### Assessment of SES

The individual SES was assessed based on family income level, occupation, education level and health insurance according to previous studies. Family income levels were defined as the family poverty to income ratio and were classified into low (≤1), middle (1–4) and high (≥4). Occupation was divided into three groups: blue collar (service, farming/forestry/fishing, precision production/craft/repair, and operator/fabricator/laborer), white collar (managerial/professional specialty, and technical/sales/administrative support), unemployed, or not in labor force (retired persons, homemakers, and students)^[19]^. Education was regrouped into less than high school, high school graduate or equivalent, and college or above. Health insurance was classified into private health insurance (any private health insurance, Medi-Gap, or single-service plan), public health insurance only (Medicare, Medicaid, State Children’s Healthcare Plan, military healthcare, Indian Health Service, State Sponsored Health Plan, or other government programme), and no health insurance. The latent class analysis (LCA), which uses multiple observed categorical variables to identify the unmeasured variable with a set of mutually exclusive latent classes, was implemented to estimate individual-level SES based on the above four variables. The PROC LCA procedure in SAS software was employed for model estimation. Three latent classes were identified in our study, which respectively represented a high, medium, and low SES according to the item-response probabilities. Model selection of latent class analysis was described in the Supplementary Digital Content Tables 1 and 2, available at: http://links.lww.com/JS9/E633.

### Assessment of lifestyle factors and other covariates

We constructed a composite healthy lifestyle score consisting of multiple domains, including cigarette smoking, alcohol consumption, physical activity and diet, which was consistent with previous NHANES studies^[16,20]^. Information on all lifestyle factors was obtained through structured questionnaires and 24-hour dietary recall. Never smoking was considered as a healthy level, defined in the questionnaire as smoking fewer than 100 cigarettes in lifetime. Alcohol consumption was defined by daily consumption of alcohol, with daily consumption of one drink or fewer for women and two drinks or fewer for men considered a healthy level^[21]^. For physical activity, we calculated weekly metabolic equivalent hours of leisure time physical activity. Participants were classified into thirds and those in the top third were considered healthy level. Dietary quality was obtained from 24-hour dietary recalls and was assessed by healthy eating index-2015 (HEI-2015) scores, which assess how well the overall diet aligns with the 2015–2020 Dietary Guidelines for Americans^[22]^. HEI-2015 scores range from 0 to 100. A healthy diet was defined as HEI-2015 score in the top two fifths of distribution^[20]^.

For each lifestyle factor, we assigned 1 point for a healthy level and 0 point for an unhealthy level. The healthy lifestyle score was calculated by summing each lifestyle factor score and ranged between 0 and 4, with higher scores indicating healthier lifestyles. Healthy lifestyle score was classified into two categories: 0–2 and 3–4 healthy behaviors. Body mass index (BMI) was not included in the overall lifestyle score due to the concern that it might act as an intermediate factor between behavioral factors and health outcomes. The obesity paradox is another concern, as many studies have found a lower risk of mortality among obese individuals^[23,24]^. In a sensitivity analysis, we included BMI in the lifestyle score. Healthy body weight was defined as a BMI of 18.5–24.9^[16]^.

Covariates were selected referring to published research, including age; sex (male or female); race (Hispanic, non-Hispanic white, non-Hispanic black, and others); marital status (not married and married/living with partner); BMI (<18.5, 18.5–24.9, 25.0–29.9, and ≥30 kg/m^2^); history of hypertension, diabetes, and cardiovascular disease (CVD).

### Ascertainment of deaths

Mortality status was retrieved from a linked mortality file provided by the National Center for Health Statistics. The file provides mortality follow-up data from the date of survey participation through 31 December 2019^[25]^. Causes of death were classified according to codes of the 10th Revision of the International Classification of Diseases. The outcomes of our study included mortality from all-cause, cancer (codes C00–C97), and non-cancer causes.

### Statistical analysis

Baseline characteristics were described across different levels of SES with weighted mean and weighted standard deviation for continuous variables as well as sample counts and weighted percentages for categorical ones. Differences among groups were tested by analysis of variance adjusted for sampling weights for continuous variables and Rao–Scott chi-square tests for categorical variables. Person years were calculated from baseline to the date of death, loss to follow-up, or 31 December 2019, whichever occurred first. All analyses accounted for the complex sample survey design of NHANES.

Cox proportional hazard regression models were used to estimate the hazard ratios (HRs) and 95% confidence intervals (CI) of outcomes associated with SES and healthy lifestyle score. Model 1 included age, sex, marital status, race, BMI; and history of hypertension, diabetes, and CVD. Model 2 additionally included the healthy lifestyle score. Mediation analysis was conducted to estimate the mediation proportion by the overall lifestyle for the association between SES and mortality by SAS macro %mediate^[26]^. In addition, we assessed the joint associations of SES and healthy lifestyle score with mortality risk. Participants were further classified into six groups based on SES (low, medium, and high) and healthy lifestyle score (0–2 and 3–4 points), with those having high SES and three to four healthy lifestyle factors serving as the reference group.

To test the robustness and potential variations across different subgroups, we conducted stratified analyses by age (<60 and ≥60), sex (men and women) and race (white and non-white participants) to explore the associations between SES and healthy lifestyles with outcomes. Age and race were each classified into two groups to increase the statistical power.

Several sensitivity analyses were conducted. First, we repeated Cox proportional hazard regression and mediation analyses by substituting SES with each socioeconomic factor (family income level, occupation, education level, and health insurance), and these factors were mutually adjusted in the models. Second, we constructed a lifestyle score that included baseline BMI. Additionally, we excluded events that occurred within the first three years of follow-up to reduce the potential reverse causation. Finally, we excluded individuals with prevalent diabetes, CVD and hypertension because both lifestyles and SES could be influenced by major chronic diseases.

Statistical analyses were performed using R (version 4.3.1) and SAS. We considered two-sided *P* values <0.05 to be significant.

## Result

### Population characteristics

A total of 2411 cancer survivors were ultimately included in this study, of whom 1279 (57.5%) were female. Descriptive characteristics of participants, stratified by SES, are presented in Table 1. Of the total participants, 626 (36.4%) had high SES, 1338 (52.2%) had medium SES, and 447 (11.4%) had low SES. Participants with low SES were more likely to be older, female, non-Hispanic White, unmarried, unemployed, less educated, have low income, and rely on public or no health insurance, with a higher prevalence of comorbidities. Moreover, unhealthy behaviors such as smoking, poor diet and insufficient leisure time physical activity were more prevalent among adults with low SES.

**Table 1:** Baseline characteristics of cancer survivors in the present study according to SES*

Characteristics	Total population	High SES	Medium SES	Low SES	*P* value
	*n* = 2411	*n* = 626	*n* = 1338	*n* = 447	
Age, %					<0.001
20–40 years	160 (8.0)	35 (6.4)	71 (6.6)	54 (17.1)	
40–60 years	524 (32.8)	178 (41.4)	245 (27.0)	101 (28.2)	
60+ years	1727(59.2)	413 (52.3)	1022 (66.4)	292 (54.7)	
Female, %	1279 (57.5)	302 (52.3)	694 (58.4)	283 (71.7)	<0.001
Race, %					<0.001
Mexican American	131 (2.1)	15 (0.7)	57 (1.7)	59 (6.3)	
Other Hispanic	102 (1.8)	20 (1.2)	45 (1.5)	37 (4.8)	
Non-Hispanic White	1776 (87.8)	522 (93.6)	1009 (88.4)	245 (73.5)	
Non-Hispanic Black	328 (5.4)	50 (2.1)	187 (5.7)	91 (12.4)	
Other race	74 (2.8)	19 (2.4)	40 (2.7)	15 (3.0)	
Marital status, %					<0.001
Married	1481 (65.5)	463 (77.4)	815 (62.6)	203 (44.1)	
Alone	777 (28.0)	131 (17.7)	450 (31.2)	196 (44.5)	
Never married or living with partner	153 (6.5)	32 (4.9)	73 (6.2)	48 (11.4)	
Family income level, %					<0.001
High	746 (42.1)	620 (99.2)	126 (11.8)	0 (0.0)	
Medium	1347 (48.8)	0 (0.0)	1171 (85.9)	176 (40.4)	
Low	318 (9.1)	6 (0.8)	41 (2.3)	271 (59.6)	
Occupation, %					<0.001
White collar	443 (27.4)	268 (51.3)	158 (16.2)	17 (4.2)	
Blue collar	277 (12.4)	33 (5.4)	199 (17.9)	45 (11.5)	
Unemployed or not in labor force	1691 (60.2)	325 (43.3)	981 (65.8)	385 (84.3)	
Education, %					<0.001
College or above	1316 (62.8)	602 (95.4)	648 (51.7)	66 (17.3)	
High school or equivalent	552 (21.9)	24 (4.6)	469 (35.2)	59 (14.8)	
Less than high school	543 (15.3)	0 (0.0)	221 (13.1)	322 (67.9)	
Health insurance, %					<0.001
Private	1432 (66.1)	507 (86.5)	903 (67.2)	22 (4.6)	
Public	828 (27.3)	119 (13.5)	352 (24.6)	357 (78.0)	
Uninsured	151 (6.6)	0 (0.0)	83 (8.2)	68 (17.4)	
Never smoking, %	1053 (44.2)	309 (50.9)	565 (41.6)	179 (35.2)	0.001
No heavy alcohol, %	2275 (92.8)	577 (90.5)	1277 (94.1)	421 (94.7)	0.021
Healthy diet, %	1254 (51.8)	399 (62.1)	685 (49.1)	170 (33.7)	<0.001
Top third of LTPA, %	511 (24.2)	220 (35.3)	237 (17.9)	54 (14.4)	<0.001
BMI, %					0.016
<18.5	43 (1.8)	14 (2.1)	17 (1.0)	12 (4.3)	
18.5–24.9	651 (28.6)	182 (30.0)	352 (26.8)	117 (30.1)	
25.0–29.9	854 (34.9)	225 (36.6)	489 (34.1)	140 (29.5)	
≥30.0	863 (34.7)	205 (31.2)	480 (38.1)	178 (36.1)	
Self-reported comorbidities					
Diabetes, %	446 (15.3)	67 (8.8)	261 (17.9)	118 (23.6)	<0.001
Hypertension, %	1375 (52.1)	301 (44.5)	795 (55.6)	279 (58.2)	<0.001
CVD, %	455 (15.9)	70 (8.5)	271 (18.4)	114 (25.3)	<0.001

* All results were survey-weighted except for counts of categorical variables; *P* value obtained from Rao–Scott chi-square tests. SES, socioeconomic status; LTPA, leisure time physical activity; BMI, body mass index; CVD, cardiovascular disease.

### Mediation analysis of lifestyle on associations of SES with mortality

During a mean follow-up of 9.0 years, 894 deaths were recorded, including 283 participants who died from cancer. After adjusting for lifestyle score and other covariates, including age, sex, race, marital status and history of comorbidities, the HRs for participants with low SES compared to those with high SES were 2.54 (1.85–3.50) for all-cause mortality, 1.92 (1.13–3.24) for cancer mortality, and 2.92 (1.96–4.33) for non-cancer mortality. The HRs without adjustment for lifestyle score were larger. Each additional healthy lifestyle factor was associated with 17% lower risks of all-cause mortality, 24% lower risks of cancer mortality and 14% lower risks of non-cancer mortality (Supplementary Digital Content Table 3, available at: http://links.lww.com/JS9/E633).

When comparing low SES with high SES, the proportion mediated by the lifestyle score was 13.5% (7.3–23.5%, *P* < 0.001) for all-cause mortality, 28.7% (10.7–57.6%, *P* < 0.001) for cancer mortality, and 8.7% (3.5–20.3%, *P* < 0.001) for non-cancer mortality (Table 2). In addition, when low SES levels were compared with high SES levels, all individual socioeconomic factors except for health insurance were associated with higher risks of all-cause, cancer and non-cancer mortality. The proportion of the association between individual socioeconomic factors and cancer mortality mediated by lifestyles was 20.0% (7.5–43.7%, *P* < 0.001) for family income level, 31.4% (11.0–62.9%, *P* < 0.001) for education attainment and 12.1% (5.0–26.5%, *P* < 0.001) for occupation (Supplementary Digital Content Table 4, available at: http://links.lww.com/JS9/E633). The results of sensitivity analyses were largely consistent with the main analysis (Supplementary Digital Content Table 5, available at: http://links.lww.com/JS9/E633).

**Table 2:** Associations of SES with mortality among US cancer survivors and mediation proportion attributed to lifestyle*

Variables	Model 1 (unadjusted for lifestyle score)	Model 2 (adjusted for lifestyle score)	Mediation proportion (%) (95% CI)	*P* value
	HR (95%CI)	HR (95%CI)		
All-cause mortality				
High SES	Ref	Ref	Ref	Ref
Medium SES	1.88 (1.49, 2.36)	1.75 (1.38, 2.22)	9.0% (4.7–16.7%)	<0.001
Low SES	2.91 (2.14, 3.96)	2.54 (1.85, 3.50)	13.5% (7.3–23.5%)	<0.001
Cancer mortality				
High SES	Ref	Ref	Ref	Ref
Medium SES	1.60 (1.11, 2.28)	1.45 (1.00, 2.09)	18.7% (7.5–39.5%)	<0.001
Low SES	2.29 (1.40, 3.76)	1.92 (1.13, 3.24)	28.7% (10.7–57.6%)	<0.001
Non-cancer mortality				
High SES	Ref	Ref	Ref	Ref
Medium SES	2.03 (1.51, 2.72)	1.92 (1.43, 2.60)	5.8% (2.2–14.4%)	0.013
Low SES	3.27 (2.24, 4.76)	2.92 (1.96, 4.33)	8.7% (3.5–20.3%)	0.009

* Socioeconomic status was generated through latent class analysis using information on family income level, occupation, education, and health insurance. Model 1 included age, sex, marital status, race, BMI, and prevalent comorbidities (including history of hypertension, diabetes, cardiovascular disease). Model 2 additionally included the healthy lifestyle score consisting of never smoking, no heavy alcohol drinking, higher physical activity level, and a higher diet quality score. All analysis included the US population and study design weights to account for the complex survey design. SES, socioeconomic status; HR, hazard ratio; CI, confidence interval.

Subgroup analyses were conducted based on age, sex, and race. Significant mediating effects of lifestyles on mortality due to SES inequality were observed in various age and race groups (Supplementary Digital Content Table 6, available at: http://links.lww.com/JS9/E633). However, the mediating effect of lifestyles was not significant among non-white participants.

### Joint analysis of lifestyle and SES with mortality

**Figure 2.**
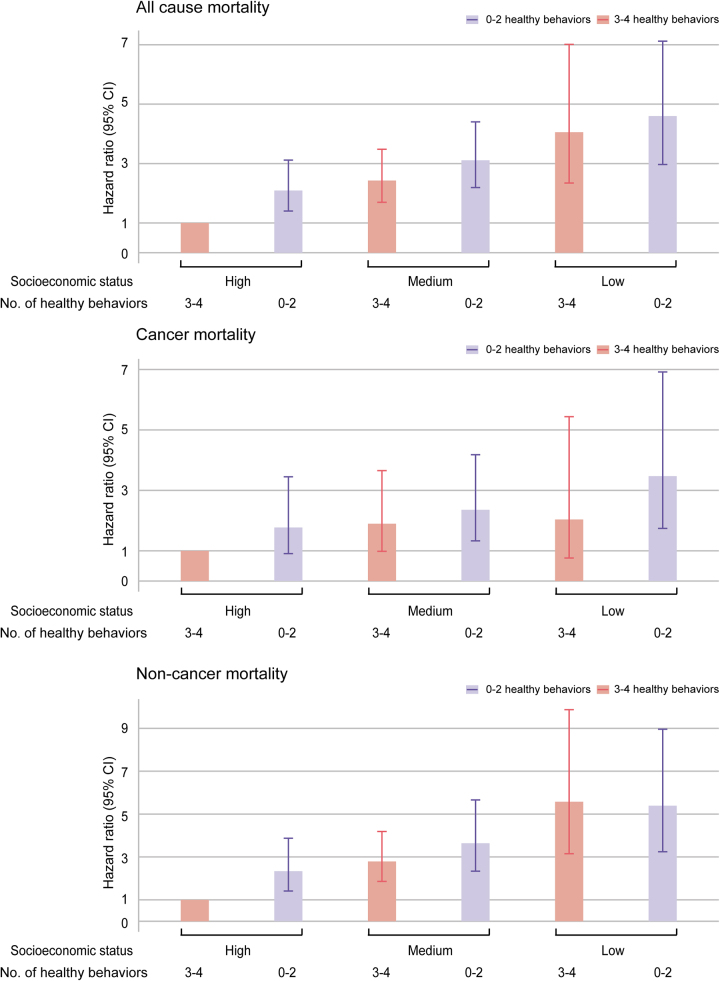
Joint associations of healthy lifestyle score and socioeconomic status with mortality among US cancer survivors. Hazard ratios were adjusted for age, sex, marital status, race, BMI and prevalent comorbidities (including history of hypertension, diabetes and cardiovascular disease). All analyses included the US population and study design weights to account for the complex survey design.

In the joint analyses, combinations of healthy lifestyles and high SES were associated with the lowest all-cause and cancer mortality risks. HRs for individuals of low SES and zero to two healthy lifestyle factors compared with those with high SES and three or four healthy lifestyle factors were 4.60 (2.97–7.12) for all-cause mortality, 3.47 (1.74–6.92) for cancer mortality, 5.39 (3.24–8.96) for non-cancer mortality (Fig. 2).

## Discussion

In the present study, we found that low SES was associated with higher mortality risks among US cancer survivors, with 8.7–28.7% of the associations mediated by lifestyle factors. Additionally, a significant joint effect of SES and lifestyle factors on mortality was observed. Compared with cancer survivors who had high SES and three to four healthy lifestyle score, those with low SES and the least healthy lifestyle score exhibited the highest risks of all-cause and cancer mortality. To our knowledge, this study is the first to examine the separate, combined and mediating relationships between SES, healthy lifestyles and mortality outcomes of cancer survivors.

Considerable evidence has discussed socioeconomic inequity in cancer patient’s survival. A retrospective cohort study with US Census Bureau data from the American Community Survey showed that lower neighborhood SES was associated with decreased survival among patients with cancer^[27]^. Byers *et al* reported that low SES was a risk factor for all-cause mortality after a diagnosis of cancer^[17]^. In addition, an umbrella review underscored that lower SES was consistently associated with a higher incidence of certain cancers, poorer prognosis, and reduced access to screening and treatment, while high SES was linked to better access to healthcare and improved outcomes^[28]^. However, previous studies usually defined SES from a single socioeconomic factor. In this research, using LCA, we created an overall SES index incorporating education, family income, health insurance and occupational position, to represent SES level. We also evaluated each individual SES variable, and obtained comparable results with previous investigations, which verified the credibility of our results. Our study confirmed that SES was a powerful determinant of mortality outcomes among cancer survivors. Therefore, exploration of possible methods to reduce socioeconomic inequity in mortality among cancer survivors is urgently needed.

Existing research indicated that SES could influence an individual’s lifestyle^[29]^. Unhealthy lifestyles including smoking, poor diet, and physical inactivity were more prevalent in low SES groups^[30,31]^. Several studies have investigated the contribution of health behaviors to socioeconomic inequity in health outcomes. A study involving two large cohorts demonstrated that maintaining healthy lifestyles appeared to counteract the adverse effects of SES on cardiovascular disease and mortality^[20]^. A systematic review of 31 studies reported that lifestyle factors accounted for approximately 20–30% of SES-related health disparities^[29]^. In addition, Hastert *et al* reported that association between area-level SES and cancer mortality among participants with no prior history of cancer at baseline is partially mediated by lifestyle factors^[32]^. However, no study has been conducted to assess the potential roles of healthy lifestyles in mitigating the total mortality risk of cancer survivors caused by SES inequality. We thus employed a mediation analysis in this study and revealed differential mediation effects of lifestyle factors across mortality outcomes among cancer survivors. Specifically, lifestyle behaviors accounted for a modest proportion of all-cause and non-cancer mortality caused by SES disparities. However, the mediation proportion was nearly 30% for cancer-related deaths. These findings indicated that lifestyle interventions may help reduce the impact of SES inequality on mortality among cancer survivors, with a potentially greater effect on cancer-specific mortality. In addition, significant joint effects of healthy lifestyles and SES with health outcomes were observed. Low SES cancer survivors with one to two healthy lifestyle score had the highest risk of all-cause and cancer mortality. Therefore, education and promotion of healthy lifestyles (e.g., smoking cessation, physical activity promotion, and dietary counseling) should be systematically integrated into primary care and the clinical management of cancer survivors, especially those with low SES.

Our study was the first to examine the complex relationships of healthy lifestyles and SES with mortality risk among cancer survivors. We constructed a composite SES variable and healthy lifestyle score to encompass the combinations of various factors. We also conducted a series of sensitivity analyses to show the robustness of the findings and evaluated individual socioeconomic and lifestyle factors. Nevertheless, we also acknowledge several limitations. First, although key personal characteristics and comorbidities were controlled, residual confounding remains possible, and causal inference cannot be established due to the observational nature of the study. However, to minimize the potential impact of reverse causality, we conducted sensitivity analyses by excluding participants who died within the first three years of follow-up after baseline assessment. Second, information on SES and lifestyle was mainly self-reported and was only measured at baseline, thus measurement errors were inevitable. Future studies with repeated measurements are preferred.

## Conclusion

In this nationally representative study of US cancer survivors, we demonstrated that socioeconomic disparities in mortality are partially mediated by modifiable lifestyle factors, with the most pronounced mediation effect observed for cancer-specific mortality. These findings suggest that promoting healthy lifestyles could mitigate a substantial proportion of socioeconomic inequities in cancer-specific outcomes. Furthermore, the joint analysis revealed that individuals with both disadvantaged SES and least healthy lifestyle scores faced the highest risks of all-cause and cancer mortality, underscoring the critical role of lifestyle interventions in reducing mortality risk among cancer survivors, particularly those from low SES populations.

## Data Availability

The datasets supporting the conclusions of this article are available in the NHANES repository (https://www.cdc.gov/nchs/nhanes/index.htm).

## Research registration unique identifying number (UIN)

As a secondary analysis of anonymized data, this study did not involve human participants.
